# Forced expression of fibroblast growth factor 21 reverses the sustained impairment of liver regeneration in hPPARα^PAC^ mice due to dysregulated bile acid synthesis

**DOI:** 10.18632/oncotarget.3531

**Published:** 2015-03-12

**Authors:** Hui-Xin Liu, Ying Hu, Samuel W. French, Frank J. Gonzalez, Yu-Jui Yvonne Wan

**Affiliations:** ^1^ Department of Medical Pathology and Laboratory Medicine, University of California, Davis, Sacramento, CA, USA; ^2^ Department of Pathology & Laboratory Medicine, Harbor-UCLA Medical Center, Torrance, CA, USA; ^3^ Laboratory of Metabolism, National Cancer Institute, National Institutes of Health, Bethesda, MD, USA

**Keywords:** nuclear receptor, metabolism, proliferation, bile acid, species differences

## Abstract

Peroxisome proliferator activated receptor α (PPARα) stimulates hepatocellular proliferation is species-specific. Activation of mouse, but not human, PPARα induces hepatocellular proliferation, hepatomegaly, and liver cancer. Here we tested the hypothesis that human and mouse PPARα affects liver regeneration differentially. PPARα-humanized mice (hPPARα^PAC^) were similar to wild type mice in responding to fasting-induced PPARα signaling. However, these mouse livers failed to regenerate in response to partial hepatectomy (PH). The liver-to-body weight ratios did not recover even 3 months after PH in hPPARα^PAC^. The mouse PPARα-mediated down-regulation of *let-7c* was absent in hPPARα^PAC^, which might partially be responsible for impaired proliferation. After PH, hPPARα^PAC^ displayed steatosis, necrosis, and inflammation mainly in periportal zone 1, which suggested bile-induced toxicity. Quantification of hepatic bile acids (BA) revealed BA overload with increased hydrophobic BA in hPPARα^PAC^. Forced FGF21 expression in partial hepatectomized hPPARα^PAC^ reduced hepatic steatosis, prevented focal necrosis, and restored liver mass. Compared to mouse PPARα, human PPARα has a reduced capacity to regulate metabolic pathways required for liver regeneration. In addition, FGF21 can compensate for the reduced ability of human PPARα in stimulating liver regeneration, which suggests the potential application of FGF21 in promoting hepatic growth in injured and steatotic livers in humans.

## INTRODUCTION

Active metabolism is required to generate energy and precursors for the biosynthesis of macromolecules used to produce cell or tissue mass to fully execute liver regeneration [1, 2]. A transient steatosis was noted in early phases of liver regeneration, and when this is disrupted, a delay in liver regeneration is noted, indicating that rapid accumulation of intracellular triglycerides may provide a crucial energy substrate for the regenerating liver [3]. Conversely, excessive accumulation of hepatic lipids is also linked to impaired liver regeneration, as demonstrated in humans as well as genetically-modified mouse models of obesity [4, 5]. Thus, lipid homeostasis plays a role in modulating liver regeneration.

Due to the important function of peroxisome proliferator-activated receptors (PPAR) in regulating lipid homeostasis, the effects of PPARα, β, and γ on liver regeneration have been studied. Notably, PPARβ regulates liver regeneration by modulating AKT and E2f signaling with PPARβ deficiency delaying normal liver regeneration in mice [1]. Disruption of hepatic PPARγ expression in mice with diet-induced hepatic steatosis resulted in significant suppression of liver regeneration [2]. Lack of PPARα also delays liver regeneration through suppression of cell cycle control, cytokine signaling, fat metabolism, and impaired Ras signaling [6, 7].

PPARα regulates not only lipid metabolism, but also cell proliferation, the latter of which is rodent specific [8]. Sustained activation of PPARα induces hepatocyte proliferation and hepatocellular carcinomas in rodents [8]. However, humans are not susceptible to the hepatocarcinogenic effects of PPARα agonists [8, 9]. It is unknown whether there exists a species difference in hepatocyte proliferation during liver regeneration. Although the strong hepatocyte proliferative effect of rodent PPARα causes liver cancer, such effective proliferative effect may be beneficial for liver regeneration. Thus, the current study examined the species-specific role of PPARα in liver regeneration by performing a 2/3 partial hepatectomy (PH) in wild-type (WT) and PPARα-humanized (hPPARα^PAC^) mice.

PPARα-regulated pathways could be important in the control of liver regeneration. For example, PPARα is a key regulator of hepatic fatty acid metabolism and can be activated by hypolipidemic drugs. Thus, activation of PPARα signaling in hepatic steatotic patients prior to liver transplantation could potentially be beneficial [10]. Stimulation of PPARα induces expression of fibroblast growth factor 21 (Fgf21), which encodes a cytokine essential for hepatic lipid oxidation and ketogenesis in the adaptive response to fasting conditions [11]. Administration of FGF21 exerts beneficial effects including reduction of adiposity, insulin resistance, dyslipidemia, and fatty liver [12, 13]. FGF21 has an effect in preventing lipopolysaccharide, acetaminophen, cerulein, and dioxin-induced toxicity and injury [11, 14-16]. Thus, it seems that PPARα-activated-FGF21 may have a role in repairing an injured liver. PPARα also regulates inflammatory pathways [10]. The IL-6-mediated STAT3 activation signaling, which is crucial for liver regeneration, is compromised in PPARα knockout mice [6]. Moreover, PPARα has a role in bile acid (BA) homeostasis [10]. Circulating blood BA levels increase after PH, and depletion of BAs decrease regeneration [17]. After PH, the remnant liver is exposed to a high flux of BAs, and regulation of BA homeostasis is essential for the normal progression of liver regeneration in rodents [17, 18]. In addition, lack of the BA receptor farnesoid x receptor (FXR) delays liver regeneration [17]. In humans, hepatocyte proliferation is commonly observed in biopsies of cholestatic livers [19]. Thus, PPARα-regulated pathways including fatty acid and BA homeostasis as well as inflammatory signaling modulate liver regeneration. However, it is unknown whether there is a species difference in those PPARα-regulated pathways during liver regeneration. The current study used hPPARα^PAC^ to compare the role of human and mouse PPARα in response to PH-induced liver regeneration.

## RESULTS

### Overnight fasting activates PPARα target genes in both WT and hPPARα^PAC^ mice

**Figure 1:**
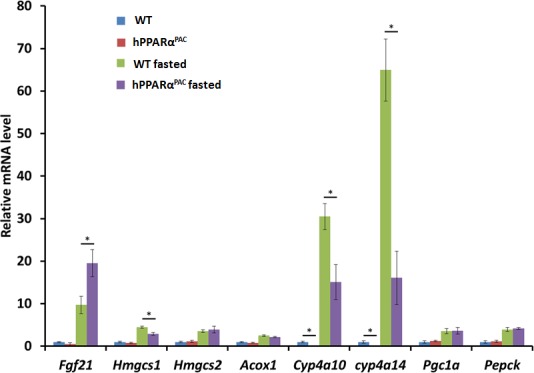
Fasting activates mouse and human PPARα target genes Wild type (WT) and PPARα-humanized (hPPARα^PAC^) mice were fasted overnight to activate PPARα signaling. The expression of hepatic PPARα target genes was studied by qPCR. Fasting induced all the PPARα target genes including *Fgf21*, *Hmgcs1* and *2*, *Acox1*, *Cyp4a10*, *Cyp4a14*, *Pgc1a*, and *Pepck* in both WT and hPPARα^PAC^ mice. All values represent mean ± standard deviation, *n* = 5; * p<0.05, student's *t* test.

Fasting activates PPARα downstream pathways in the mouse liver resulting in increased fatty acid catabolism [11]. Thus, hPPARα^PAC^ mice were subjected to fasting to determine whether the fasting-induced PPARα signaling activation is conserved with the human receptor. Overnight-fasting induced mRNAs encoded by the PPARα target genes Fgf21, HMG-CoA synthase (*Hmgcs*) 1 and 2, acyl-CoA oxidase 1 (*Acox1*), cytochrome P450 4a10 (*Cyp4a10*), *Cyp4a14*, peroxisome proliferator-activated receptor co-activator protein 1α (*Pgc1a*), and phosphoenolpyruvate carboxykinase (*Pepck*) in WT mice (Fig. 1). Fasting induced higher *Fgf21* (18 vs. 9.8) and lower *Hmgcs1* (2.8 vs.4.3), *Cyp4a10* (12 vs. 27), and *Cyp4a14* (17 vs. 61) mRNA levels in hPPARα^PAC^ than in WT mice, while fasting-induced *Hmgcs2*, *Acox1*, *Pgc1a*, and *Pepck* mRNA levels were comparable in the two mouse lines. These data indicate that human PPARα has a functional response to fasting, which is consistent with published findings [8].

### Reduced liver growth in hPPARα^PAC^ mice after PH

**Figure 2:**
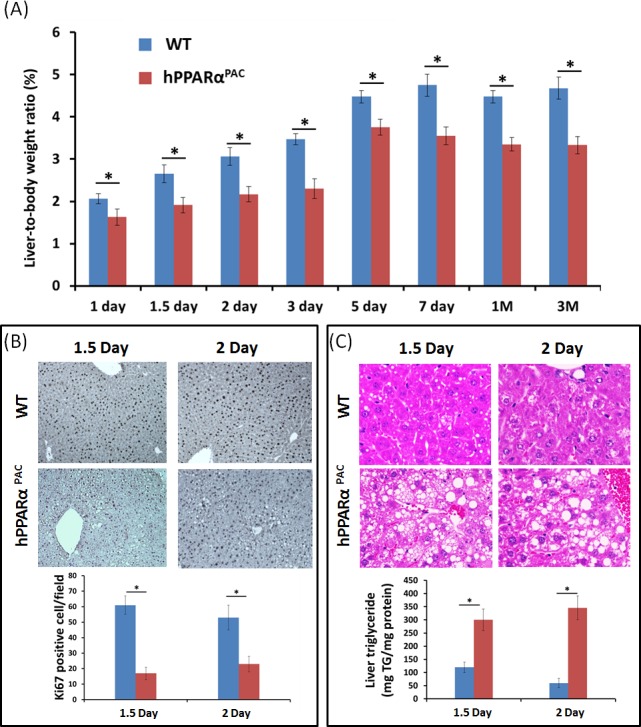
PH-induced liver growth is suppressed in hPPARα^PAC^ mice Wild type (WT) and PPARα-humanized (hPPARα^PAC^) mice were subjected to PH and killed 0 day to 3 months after surgery. Liver-to-body weight ratios were recorded. The data indicated that liver regeneration was not completed in hPPARα^PAC^ mice 3 months after the surgery (A). Representative Ki67 staining (10×) of mouse livers after PH. The number of proliferative cells was significantly less in hPPARα^PAC^ than WT mice (B). Representative images of H&E staining (40×) of liver sections of mice that received PH indicated that hPPARα^PAC^ mice had fat deposition. Hepatic triglyceride levels 1.5 and 2 days after PH indicated elevated triglyceride in hPPARα^PAC^ livers after PH (C). All values represent mean ± standard deviation, *n* = 5; * p<0.05, student's *t* test.

**Figure 3:**
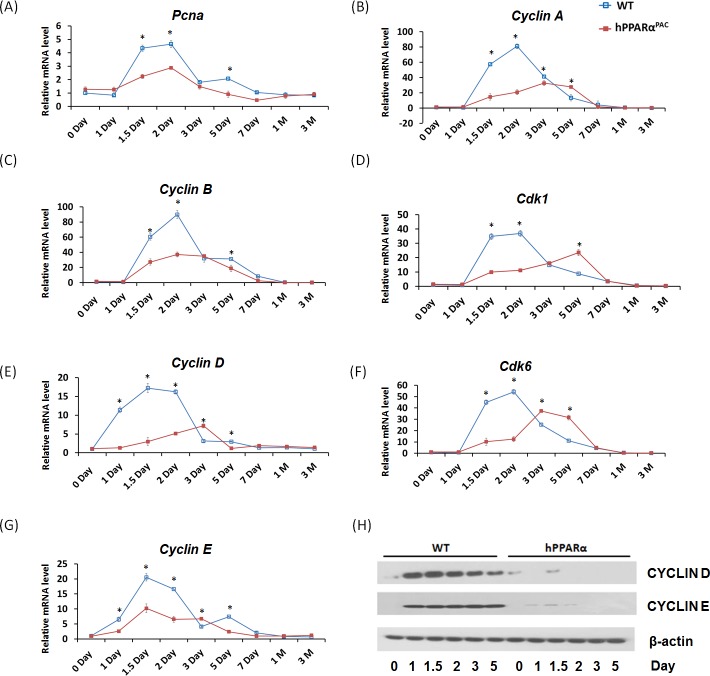
The induction of hepatic cell cycle genes is diminished in hPPARα^PAC^ mice after PH Experiments were performed based on the description in Figure legend 2. Hepatic gene expression of *Pcna*, *Cyclin A*, *Cyclin B*/*Cdk1*, *Cyclin D*/*Cdk6*, and *Cyclin E* was studied by qPCR in WT and hPPARα^PAC^ mouse livers after PH (A-G). Western blot analysis of CYCLIN D and E protein levels in WT and hPPARα^PAC^ livers after PH (H). All values represent mean ± standard deviation, *n* = 5; * p<0.05, student's *t* test.

**Figure 4:**
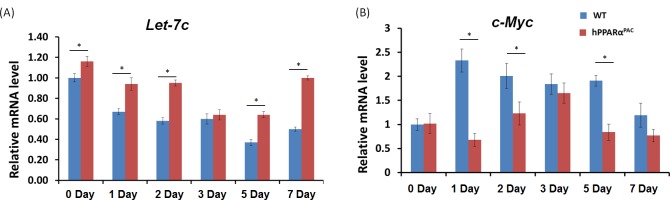
Hepatic expression levels of *let-7c* miRNA and *c-Myc* mRNA in WT and hPPARα^PAC^ mice after PH Experiments were performed based on the description in Figure legend 2. The expression of *let-7c* (A) and *c-Myc* (B) was studied by qPCR. A significantly higher expression of *let-7c* was found in hPPARα^PAC^ mice at basal level compared with that in WT mice. After PH, the expression of *let-7c* decreased in both genotypes of mice. However, the decreased levels were significantly less in hPPARα^PAC^ than WT mice in most studied time points. Accordingly, higher induction of *c-Myc* mRNA at day 1, 2, and 5 was found in WT than hPPARα^PAC^ livers. All values represent mean ± standard deviation, *n* = 5; * p<0.05, student's *t* test.

PH was performed in WT and hPPARα^PAC^ mice, and livers were collected on 1 day up to 3 months post-surgery. The liver-to-body weight ratio of WT and hPPARα^PAC^ mice (without surgery) ranged from 4.2-4.5% and no statistical difference was found between the groups (data not shown). After PH, the liver-to-body weight ratio restored within 7 days in WT mice, whereas in hPPARα^PAC^ mice, the liver-to-body weight ratios were reduced at all studied time points compared to WT controls. The liver-to-body weight ratio was 3.6% from 7 days to 3 months after PH in hPPARα^PAC^ mice indicating that these mice failed to restore their original liver mass and normal liver regeneration was disrupted (Fig. 2A). WT livers, compared with hPPARα^PAC^ livers, exhibited greater hepatocellular proliferation, as shown by increased numbers of Ki67-positive hepatocytes after PH (Fig. 2B). Hematoxylin and eosin (H&E) staining revealed transient accumulation of hepatic lipids in WT mice 1.5 days after PH, which is likely essential for liver regeneration [3]. Two days after PH, lipid accumulation was diminished in WT livers (Fig. 2C). Representative images of hPPARα^PAC^ liver sections harvested 1.5 and 2 days post-PH indicate that hPPARα^PAC^ mice had lipid deposition (Fig. 2C). hPPARα^PAC^ mice also showed 3 to 7-fold increase in hepatic triglyceride levels 1.5 and 2 days after PH (Fig. 2C). Furthermore, the increased cell proliferation found in WT mice 1.5-2 day after PH were accompanied by higher expression levels of proliferating cell nuclear antigen (*Pcna*), *Cyclin A*, *Cyclin B*/Cyclin-dependent kinase (*Cdk*) 1 complex, *Cyclin D*/*Cdk6* complex, and *Cyclin E* mRNAs (Fig. 3A-G). However, such inductions were delayed and reduced in hPPARα^PAC^ mouse livers. Moreover, Western blots indicated that CYCLIN D and E proteins were induced after PH in WT mouse livers, but not in hPPARα^PAC^ livers (Fig. 3H). In addition, the expression of PPARα target *let-7c*, which promotes cell cycle arrest by targeting *c-Myc* mRNA [20], was studied to compare the differences between mouse and human PPARα in regulating liver regeneration. A temporal pattern of down-regulated *let-7c* was observed in regenerating WT mice. At most studied time points, except 3 days after PH, *let-7c* levels were higher in hPPARα^PAC^ than WT mouse livers (Fig. 4A). Accordingly, *c-Myc* mRNA levels were lower in hPPARα^PAC^ than WT mouse livers 1, 2, and 5 days after PH (Fig. 4B).

### Diminished FGF21 expression and its downstream lipid homeostasis target genes in hPPARα^PAC^ mouse livers after PH

**Figure 5:**
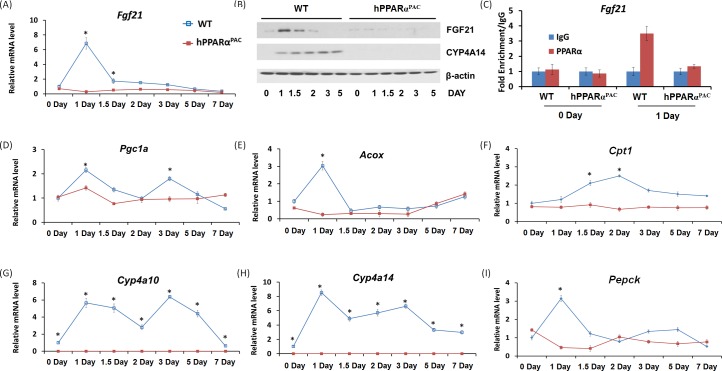
The induction of PPARα target FGF21 is diminished in PPARα-humanized mice after PH Experiments were performed based on the description in Figure legend 2. PH was performed in WT and hPPARα^PAC^ mice and hepatic gene expression of *Fgf21* was studied using qPCR (A). Western blot analysis indicated that FGF21 protein level peaked 1 day after PH in WT livers, but not in hPPARα^PAC^. The induction of CYP4A14 was found in WT mice after PH, but such induction was absent in hPPARα^PAC^ livers after PH (B). Chromatin immunoprecipitation assays were performed using liver tissues (n=3) from WT and hPPARα^PAC^ mice 0 and 1 day after PH with either PPARα or negative control IgG. The purified DNA fragments were amplified using primers specific for the *Fgf21* promoter (C). PH was performed in WT and hPPARα^PAC^ mice and hepatic gene expression of *Pgc1a*, *Acox*, *Cpt1*, *Cyp4a10, Cyp4a14* and *Pepck* were studied using qPCR (D-I). All values represent mean ± standard deviation, *n* = 5; * p<0.05, student's *t* test.

FGF21 plays a key role in regulating fatty acid oxidation, triglyceride clearance, and ketogenesis in the liver [11]. *Fgf21* mRNA level was induced and peaked in WT mice 1 day after PH. In contrast, *Fgf21* mRNA was not detected in hPPARα^PAC^ mouse livers after PH (Fig. 5A). Consistent finding was noted at the protein level (Fig. 5B). In addition, the induction of CYP4A14 protein was observed in WT mice, but not detectable in hPPARα^PAC^ livers after PH (Fig. 3B). Moreover, the binding of PPARα in the promoter region of the *Fgf21* gene was enriched in WT mouse regenerating livers, but not in hPPARα^PAC^ mice as demonstrated by ChIP-qPCR (Fig. 5C). FGF21 induces PGC1α and its target genes in response to starvation [21]. To further assess the role of FGF21 in liver regeneration, the expression of FGF21 target genes involved in lipid homeostasis (*Pgc1a*, *Acox1*, *Cpt1*, *Cyp4a14*, *Cyp4a10*, *Pepck*) was studied (Fig. 5D-I). In WT mice, the induction of these mRNAs correlated with the observed induction of *Fgf21* mRNA 1 day post-PH. There was no induction of the aforementioned genes in hPPARα^PAC^ livers up to 7 days after PH. *Cyp4a10* and *Cyp4a14* mRNA was not detectable in hPPARα^PAC^ mouse livers.

### Liver pathology

**Figure 6:**
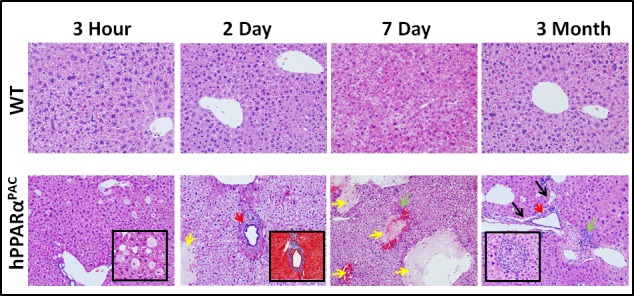
Liver injury in regenerating hPPARα^PAC^ mice Experiments were performed based on the description in Figure legend 2. Representative images of H&E staining (10×) of liver sections of mice that received PH indicated that WT had normal histology post-surgery, but regenerating hPPARα^PAC^ mice had zone 1 periportal injury. Focal necrosis indicated by yellow arrows; dilated bile duct indicated by red arrows; dilated lymphatic indicated by black arrows; interface hepatitis indicated by green arrows. Inserts show liver injury in greater detail including vacuolation and focal necrosis of hepatocytes (3 hours, 40×), periportal fibrosis (Masson's trichrome staining, 10×, 2 days), and interface hepatitis (3 months, 40×) in hPPARα^PAC^ mice.

hPPARα^PAC^ mouse livers had pathological features of vacuolation and focal necrosis 3 hours post-PH. Steatosis, dilate bile duct, dilated lymphatic, and duct metaplasia with periportal fibrosis were noted 2 days after PH. Interface hepatitis where lymphocytes invaded from the portal tract and mild steatohepatitis were notice from 7 days and 3 months post-PH (Fig. 6). These morphological features indicated zone 1 periportal injury. The post-PH injury observed in hPPARα^PAC^ mice suggested bile-induced toxicity [18], thus, hepatic BAs were quantified.

It is important to note that the injury was not due to surgery itself because none of WT mice had liver pathology after the surgery.

### Quantification of hepatic BA and study the expression of genes regulate BA homeostasis

**Figure 7:**
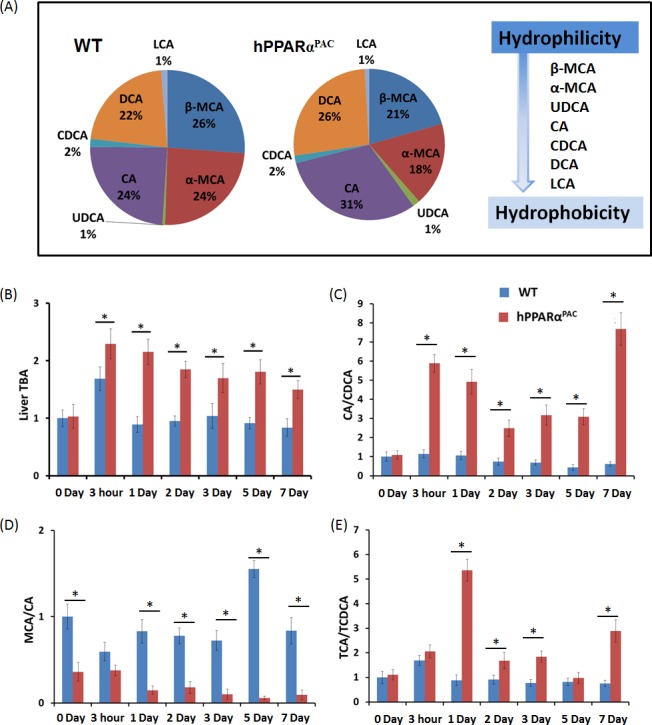
Dysregulated bile acid homeostasis in regenerating hPPARα^PAC^ mice Experiments were performed based on the description in Figure legend 2. Quantification of individual hepatic BAs in WT and hPPARα^PAC^ before surgery (A), and calculated TBA, CA/CDCA, MCA/CA, and TCA/TCDCA ratios from livers that received PH on the basis of mass spectrometry analysis (B-E). All values represent mean ± standard deviation, *n* = 5.

The basal hepatic total bile acid (TBA) level was similar between WT and hPPARα^PAC^ mice, but the composition of BA was different (Fig. 7A, B). The levels of primary BAs including CA, CDCA, MCA, UDCA were higher in WT than hPPARα^PAC^ mice (Fig. 7A). After PH, TBA level was transiently increased within 3 hours of PH and returned to the basal level 1 day later (Fig. 7B). In contrast to WT mice, sustained increase in TBA was noted in hPPARα^PAC^ mice post-PH (Fig. 7B). The ratio of CA to CDCA, frequently found to be higher in patients who have inflammation or cholestasis [22, 23], was calculated during liver regeneration in both genotypes. The CA/CDCA ratio substantially increased in regenerating hPPARα^PAC^ mice (Fig. 7C). The MCA/CA ratio was lower in hPPARα^PAC^ than WT mice also suggested increased hydrophobicity after PH in hPPARα^PAC^ mice (Fig. 7D) [18]. The increased ratio of TCA to TCDCA, which has been implicated in CCl_4_-induced acute liver injury in mice [24], was also calculated, and the ratio was higher in hPPARα^PAC^ than WT mice 1, 2, 3, and 7 days post-PH (Fig. 7E).

**Figure 8:**
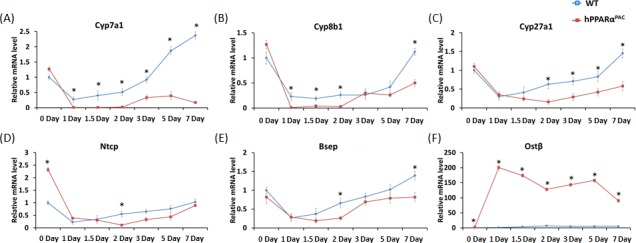
Dysregulated expression of genes involved in bile acid homeostasis in regenerating hPPARαmice Experiments were performed based on the description in Figure legend 2. Hepatic gene expression of *Cyp7a1*, *Cyp8b1*, *Cyp27a1, Ntcp*, *Bsep*, and *Ostb*, were studied using real-time PCR in regenerating WT and hPPARα^PAC^ mouse livers (A-F). All values represent mean ± standard deviation, *n* = 5; * p<0.05, student's *t* test.

The expressions of genes encoding proteins that regulate BA homeostasis were then examined. Before PH, hPPARα^PAC^ mice had higher mRNA levels of solute carrier family 10 member 1 (*Ntcp*), and organic solute transporter beta (*Ostb*), but similar mRNA levels of *Bsep* and *Cyp7a1*/*8b1*/*27a1*, as compared to WT mice (Fig. 8A-C). In addition, small heterodimer partner (*Shp*) mRNA level was more than two folds higher in hPPARα^PAC^ mice compared to WT mice (data not shown). After PH, the mRNA levels of *Cyp7a1*/*8b1*/*27a1* were suppressed in both genotypes. However the mRNA levels of *Cyp7a1*/*8b1* were barely detectable in hPPARα^PAC^ 1-2 day after PH (Fig. 8A, B). The mRNA levels of *Ntcp* and *Bsep* showed similar expression profile in both genotypes, but levels were lower in hPPARα^PAC^ than WT mice 2 days post-PH (Fig. 8D-E). Moreover, *Ostb* mRNA level was robustly induced by 62-127 fold in hPPARα^PAC^ mice compared with 2-7 fold induction in WT mice post-PH (Fig. 8F).

### Dysregulation of inflammatory and fibrotic genes in hPPARα^PAC^ livers after PH

**Figure 9:**
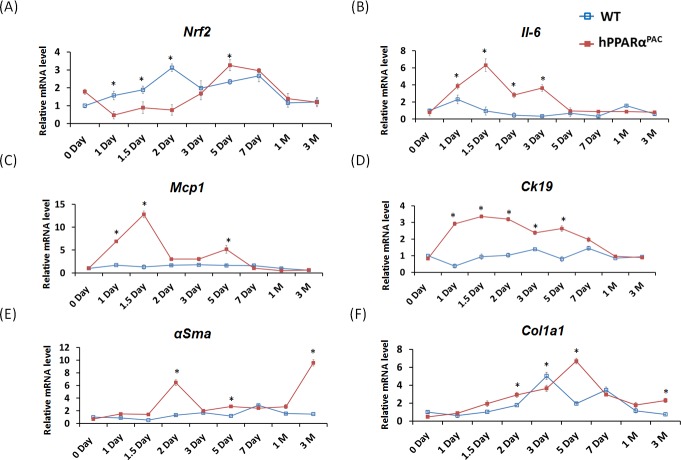
The expression profiles of hepatic genes Experiment was performed based on the description in Figure legend 2. Hepatic gene expression of *Nrf2*, *IL-6*, *MCP1*, *CK19*, *αSma* and *Col1a1* were studied using real-time PCR in regenerating WT and hPPARα^PAC^ mouse livers (A-F). All values represent mean ± standard deviation, *n* = 5; * p<0.05, student's *t* test.

Inflammatory signaling and fibrotic response were studied due to the occurring pathology in hPPARα^PAC^ mice. The nuclear factor erythroid 2-related factor (*Nrf2*) was studied for its protective function against oxidative stress. During normal liver regeneration, *Nrf2* was induced and peaked 2 days after PH in WT mice. The induction of *Nrf2* was absent in hPPARα^PAC^ mice during the first 2 days after PH, indicating impaired regulation of anti-oxidative stress by human PPARα (Fig. 9A). Pro-inflammatory genes including interleukin 6 (*Il-6*), and membrane cofactor protein 1 (*Mcp1*) were highly induced in hPPARα^PAC^ mice 1.5 days post-PH in comparison with WT counterparts (Fig. 9B-C). The expression levels of cytokeratin-19 (*Ck19*), which typically expressed in hepatobiliary tracts, were minimally changed in WT, but markedly elevated in the hPPARα^PAC^ mice post-PH. The expression levels of fibrosis marker α-smooth muscle actin (*αSma*) and collagen type I, α 1 (*Col1a1*) also showed higher induction in hPPARα^PAC^ than WT mice after PH (Fig. 9E-F).

### Adenoviral Fgf21 infusion rescues liver regeneration in hPPARα^PAC^ mice

**Figure 10:**
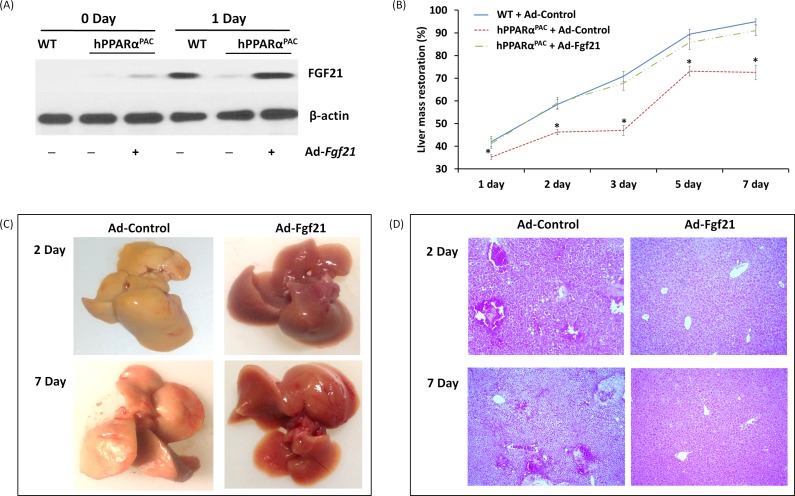
Adenoviral delivery of Fgf21 reversed the impaired liver regeneration in hPPARα^PAC^ mice Adenovirus-mediated expression of FGF21 in hPPARα^PAC^ mouse livers was accomplished via tail vein injection of Ad-Fgf21 or control vector (Ad-Control) followed by PH surgery. Western blot analysis revealed restoration of FGF21 protein levels in hPPARα^PAC^ mice was similar to that in WT (A). Liver-to-body weight ratios were recorded (B). Representative images of hPPARα^PAC^ livers with Ad-control or Ad-Fgf21 injection harvested 2 and 7 days after PH (C). Representative images of H&E staining (10×) of hPPARα^PAC^ liver sections with and without Ad-Fgf21 injection harvested 2 and 7 days after PH (D). All values represent mean ± standard deviation, *n* = 5; * p<0.05, student's *t* test, hPPARα^PAC^ with Ad-control *vs* WT with Ad-Control.

**Figure 11:**
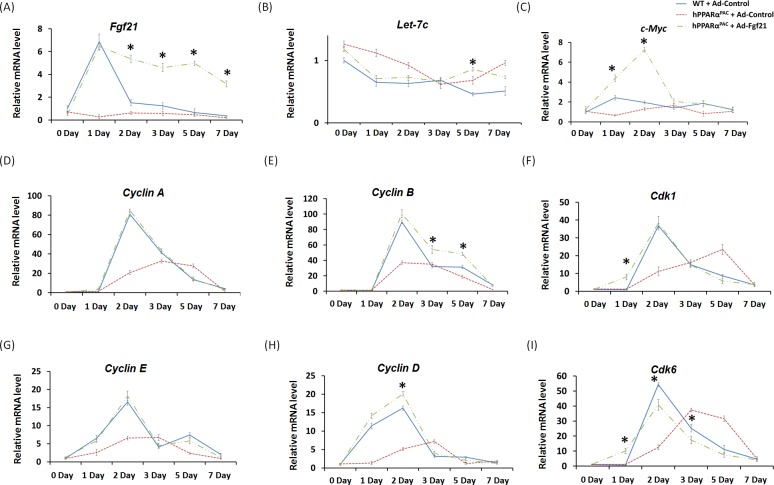
Adenoviral delivery of Fgf21 reversed the impaired cell cycle gene expressions in regenerating hPPARα^PAC^ mice Experiment was performed based on the description in Figure legend 10. Hepatic gene expression of *Fgf21*, *let-7c*, *c-Myc*, and cell cycle genes including *Cyclin A, B, D, E* as well as *Cdk1*, and *Cdk6* were studied using real-time PCR in regenerating WT mice, hPPARα^PAC^ mice, and hPPARα^PAC^ mice with Ad-Fgf21 injection (A-I). All values represent mean ± standard deviation, *n* = 5; * p<0.05, student's *t* test, hPPARα^PAC^ with Ad-Fgf21 *vs* WT with Ad-Control.

In response to fasting, the induction of FGF21 was greater in hPPARα^PAC^ than WT mice (Fig. 1). However, in response to liver resection, FGF21 mRNA and protein (Fig. 5) and its downstream targets that regulate lipid homeostasis (Fig. 5) were not induced in hPPARα^PAC^ mouse livers. These differences point to potential species differences in human and mouse PPARα signaling on the FGF21 metabolic pathway in the context of liver regeneration. An adenoviral vector expressing mouse Fgf21 (Ad-Fgf21) was used to express FGF21 in hPPARα^PAC^ mouse livers. This construct produced significant expression of FGF21 protein within 1 day after the injection (Fig. 10A). hPPARα^PAC^ mice infused with FGF21 had similar liver-to-body weight ratios as the WT mice during the course of liver regeneration (Fig. 10B). In addition, Ad-Fgf21-injected hPPARα^PAC^ mice no longer had steatosis and focal necrosis (Fig. 10C-D). Moreover, they displayed increased expression of Fgf21, which was sustained 7 days post-pH (Fig. 11A). Forced FGF21 expression also restored the *Let-7c*/*c-Myc* pathway 1-2 days post-PH in hPPARα^PAC^ mouse livers (Fig. 11B, C). In addition, expression of FGF21 in hPPARα^PAC^ mice led to the coordinated up-regulation of *Cyclin A*, *Cyclin B*/*Cdk1* complex, *Cyclin E*, and *Cyclin D*/*Cdk6* complex (Fig. 11D-I).

**Figure 12:**
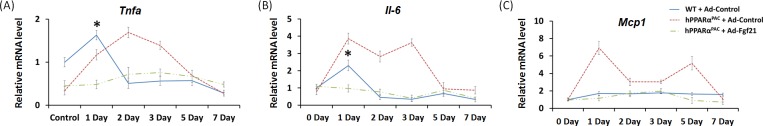
Adenoviral delivery of Fgf21 reversed the dysregulated expression of genes that are involved in pro-inflammation in regenerating hPPARα^PAC^ mice Experiment was performed based on the description in Figure legend 10. Hepatic gene expression of pro-inflammation genes (*Tnfα*, *Il-6*, *Mcp1*) were studied using real-time PCR in regenerating WT mice and hPPARα^PAC^ mice with Ad-Control, and hPPARα^PAC^ mice with Ad-Fgf21 injection (A-C). All values represent mean ± standard deviation, *n* = 5; * p<0.05, student's *t* test, hPPARα^PAC^ with Ad-Fgf21 *vs* WT with Ad-Control.

**Figure 13:**
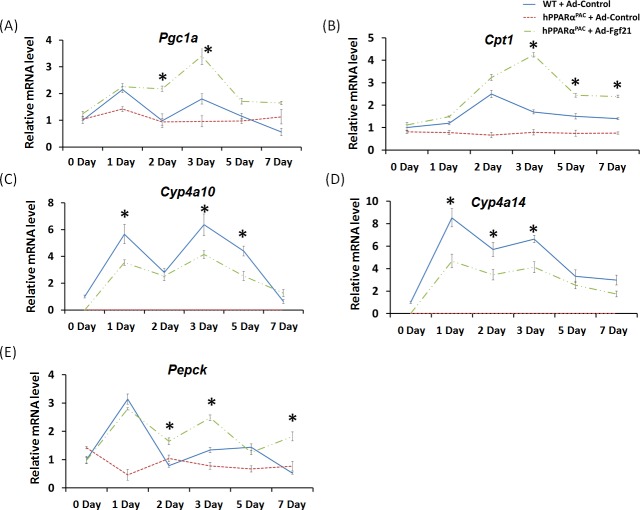
Adenoviral delivery of Fgf21 reversed the dysregulated expression of genes that are involved in lipid homeostasis in regenerating hPPARα^PAC^ mice Experiment was performed based on the description in Figure legend 10. Hepatic gene expression of PPARα target genes involved in lipid homeostasis (*Pgc1a*, *Cpt1*, *Cyp4a10*, *Cyp4a14*, *Pepck*) were studied using real-time PCR in regenerating WT mice and hPPARα^PAC^ mice with Ad-Control, and hPPARα^PAC^ mice with Ad-Fgf21 injection (A-E). All values represent mean ± standard deviation, *n* = 5; * p<0.05, student's *t* test, hPPARα^PAC^ with Ad-Fgf21 *vs* WT with Ad-Control.

**Figure 14:**
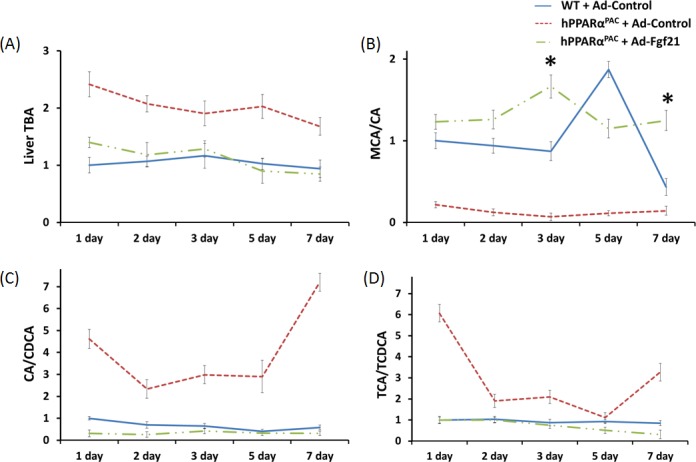
Adenoviral delivery of Fgf21 reversed the dysregulated bile acid homeostasis in regenerating hPPARα^PAC^ mice Experiment was performed based on the description in Figure legend 10. Quantification of TBA, CA/CDCA, MCA/CA, and TCA/TCDCA ratio from livers that received PH on the basis of mass spectrometry analysis (A-D). All values represent mean ± standard deviation, *n* = 5; * p<0.05, student's *t* test, hPPARα^PAC^ with Ad-Fgf21 *vs* WT with Ad-Control.

Forced FGF21 expression reduced the mRNA levels of *Tnfa*, *Il-6*, and *Mcp1* in hPPARα^PAC^ mouse livers that had PH (Fig. 12). Consistent with the phenotype, forced FGF21 expression also induced the expression of *Pgc1a*, *Cpt1*, *Cyp4a10*, *Cyp4a14*, and *Pepck* genes suggesting normalization of lipid homeostasis in hPPARα^PAC^ mice (Fig. 13). BA quantification showed normalized hepatic TBA, MCA/CA, CA/CDCA, and TCA/TCDCA values in hPPARα^PAC^ mouse livers with Ad-Fgf21infusion (Fig. 14).

## DISCUSSION

The current study demonstrated that hPPARα^PAC^ mice exhibit reduced hepatocyte proliferative capability during liver regeneration in comparison with WT mice. The presented data showed that human PPARα-mediated signaling that controls liver regeneration was less effective than that of mouse PPARα. PH caused persistent steatosis, inflammation, and necrosis, which was associated with dysregulated lipid and BA homeostasis in hPPARα^PAC^ mice. Thus, in response to liver regeneration, hPPARα is not as effective as mouse PPARα in regulating lipid metabolism as well as hepatocyte proliferation. Metabolism, which is mainly controlled by the liver, is about 7 times faster in mice than humans [25]. Liver regeneration, which can be completed within 7-10 days in mice, takes about 60-90 days to complete in humans [26]. Thus, it seems that the metabolic rate and proliferative capability are correlated, and that the species difference of PPARα may account for such difference. Because overexpression of FGF21 could restore the normal progression of liver regeneration in hPPARα^PAC^ mice, FGF21 appears to not only repair injury, but also compensate for the reduced ability of human PPARα to hasten liver regeneration. These findings suggest that FGF21 infusion would be of therapeutic value to improve the outcome of liver transplantation and liver disease in humans.

The hPPARα^PAC^ mice used in the current study were generated with a P1 phage artificial chromosome clone containing the complete human PPARα gene including the 5′ and 3′ flanking sequences [8]. Thus, the strategy used allowed the WT and hPPARα^PAC^ mice to have the same PPARα tissue distribution pattern, and the levels of PPARα are comparable in liver, brown adipose tissue, kidney, heart, intestine, lung, etc. [8]. Moreover, hPPARα^PAC^ and WT mice respond to fasting as well as fenofibrate in a similar manner, which include peroxisome proliferation, reduction of serum triglycerides, and induction of genes encoding enzymes involved in fatty acid metabolism in liver, kidney, and heart. Thus, the observed difference was not due to differential tissue distribution pattern or difference in expression level.

Published data suggested that the species difference of PPARα might be due to human livers having 10-fold lower PPARα expression level compared to mouse livers [27]. However, others showed that the level of PPARα mRNA was similar between human and mouse livers as well as primary hepatocytes [28]. It is important to note that PPARα is regulated by the circadian cycle [29]. Thus, it is difficult to compare the expression level of PPARα in human livers without knowing the time of liver harvesting and the expression at the protein level. Microarray data showed that activation of PPARα regulates a mostly divergent set of genes in mouse and human hepatocytes although the key role of PPARα as the master regulator of hepatic lipid metabolism is generally well-conserved between mouse and human [8]. Consistently, the data presented in current study showed that PPARα target genes, such as Fgf21, can be induced to different levels depending on the challenges and species. In response to fasting, human PPARα-induced Fgf21 was higher than mouse PPARα, whereas in response to liver resection, human PPARα was not able to induce Fgf21 at all. The binding data indicated that the lack of induction of FGF21 was associated with lack of PPARα binding to the Fgf21 gene in hPPARα^PAC^ mice after PH. Such differences might be due to the presence of different endogenous ligands, which have different binding affinity to human and mouse PPARα, in response to the specific challenges.

FGF21 is a hepatokine that acts as a global starvation signal. As a key metabolic regulator, FGF21 controls glucose and lipid homeostasis [30-33]. Therapeutic administration of recombinant FGF21 exerts a variety of beneficial effects in rodents and nonhuman primates, including reduction of adiposity and alleviation of hyperglycemia, insulin resistance, dyslipidemia, and fatty liver disease [21]. FGF21 deficiency increases the susceptibility of mice to cerulein-induced pancreatitis and toxicity of sepsis, as well as acetaminophen-induced liver injury, suggesting a potentially protective effect of FGF21 against acute organ injury [11, 14-16]. Replenishment of FGF21 protects acute liver failure from acetaminophen-inducted hepatic ROS accumulation and reverses the abolishment of antioxidant gene expression by increasing PGC1α [16]. FGF21 regulates energy homeostasis through activation of AMPK and SIRT1 in mice [34]. SIRT1 is implicated in liver regeneration by regulating bile acid metabolism [35]. Published data suggested that FGF21 regulates AMPK through interaction with between ERK1/2 and LKB1 [34], and LKB1/AMPK activation plays a critical role in controlling *Cyclin D* and *Cyclin A* expression [36]. Moreover, ERK1/2 signaling promotes cell proliferation by regulating cell cycle progression during liver regeneration in mice [37]. FGF21 may regulate liver regeneration through these pathways. Additional studies are needed to understand the role of FGF21 in tissue repair and liver regeneration. In addition to PPARα, FGF21 can be regulated by other nuclear receptors that include the bile acid receptor FXR, retinoic acid receptor, and receptor-related orphan receptor α [30-33]. It is also possible that these nuclear receptor-mediated signaling pathways were altered in hPPARα^PAC^ mice after PH, a possibility that needs to be explored.

One distinct difference between human and mouse PPARα is their proliferative effect. Fenofibrate induces hepatocyte proliferation in WT mice, but not in hPPARα^PAC^ mice, and a differential regulation of oncogenic *let-7c* by PPARα accounts for the species difference in proliferation [20]. *Let-7c* is markedly reduced in highly proliferative cancer cells and overexpression of *let-7c* inhibits cancer cell growth [20]. *Let-7c* repression is mouse PPARα-dependent [20]. Notably, mouse, but not human, PPARα up-regulates *c-Myc*-induced cell cycle gene expression through *let-7c* inhibition [20]. The expression of *let-7c* levels is reduced 2 hours after PH in rat, suggesting that *let-7c* repression is essential for cell cycle genes induction [38]. The diminished inhibition of *let-7c* and reduced cell cycle gene expressions in regenerating hPPARα^PAC^ mouse livers support the view that an inherent difference exists between mouse and human PPARα in regulating cell proliferation. Forced expression of FGF21 normalized the expression pattern of *let-7c* and *c-Myc* 1-2 days post-PH in hPPARα^PAC^ mouse livers. The relationship between FGF21-regulated metabolism and *let-7c*/*c-Myc*-regulated cell proliferation remains to be established.

PPARα is a major regulator of BA synthesis [39], and high BAs were persistently present in regenerating hPPARα^PAC^ livers. In contrast, WT mice only had a modest and transient increase in hepatic TBA after PH. Dysregulated BA homeostasis was likely the direct cause of liver injury because sustained BA dysregulation was found in regenerating hPPARα^PAC^ mouse livers, which have hepatic injury focused around the periportal area. In addition, the composition of BAs was altered with increased hydrophobicity found in hPPARα^PAC^ mice. Fine-tuning BA levels is essential during liver regeneration to protect hepatocytes from BA-induced toxicity while allowing sufficient BA signaling to stimulate regeneration [17, 18]. PPARα activation in regulating CYP7A1 and BA homeostasis is controversial. Stimulation of PPARα by fibrates represses mRNA levels and enzyme activity of human CYP7A1, whereas activation of PPARα induces mouse *Cyp7a1* transcriptional activity [39]. The differential regulation of the *Cyp7a1* by PPARα in humans and mice could be a novel species-specific effect. The basal levels of *Cyp7a1*/*8b1*/*27a1* expression were similar in both genotypes of mice. However, liver injury and inflammation can inhibit *Cyp7a1* gene expression [40], which might in part responsible for reduced *Cyp7a1* expression in hPPARα^PAC^ mouse livers after PH. *Ostb* mRNA is relatively abundant in human liver, but not in mouse liver and expression of *Ostb* is mainly positively regulated by BAs via the FXR [41]. Enhanced hepatic *Ostb* mRNA and protein is found in patients who have primary biliary cirrhosis and in animal model of cholestasis [41]. Induction of *Ostb* increases bile salt flux and reduces the accumulation of intracellular bile salts [42]. A dramatic induction of *Ostb* mRNA in hPPARα^PAC^ livers after PH suggested increased bile salt burden post-surgery. Enhanced BA levels which resulted from dysregulated expression of genes involved in BA synthesis and transport could also account for periportal pathology found in hPPARα^PAC^ livers after PH.

In conclusion, liver regeneration is impaired in hPPARα^PAC^ mice, which may be partially due to an over-accumulation of lipids and BA. Compared to murine PPARα, human PPARα is ineffective in regulating lipid and BA metabolism in response to PH. However, forced expression of FGF21 can reverse this deleterious condition and restore normal liver regeneration programs in hPPARα^PAC^ mice. This finding indicates the importance of FGF21 in liver regeneration and suggests its potential application in promoting hepatic growth in injured and steatotic livers in humans.

## MATERIALS AND METHODS

### Animal

Male WT and hPPARα^PAC^ mice [8] were used for this study. The hPPARα^PAC^ mice, on a *Ppara*-null background, express the human PPARα gene, and were characterized previously [8]. Mice, 3- to 5-month-old were housed in steel microisolator cages at 22°C with a 12-hr light/dark cycle. Food and water were provided *ad libitum* throughout the study with the exception of fasting experiments. Standard 2/3 PH was performed using the procedure described previously [1, 43, 44]. Mice were killed 3 hours, 1, 1.5, 2, 3, 5, 7 days and 1, 3 months after PH. At least 5 mice were used to study each time point. The liver and body weights at the time of death were recorded to calculate liver-to-body weight ratios. The results obtained were the mean of at least five mice per studied time point. A section of each liver sample was fixed in 10% formalin, embedded in paraffin, and stained for histological analysis. All animal experiments were conducted in accordance with the National Institutes of Health Guide for the Care and Use of Laboratory Animals under protocols approved by the Institutional Animal Care and Use Committee of the University of California, Davis.

### Ki67 immunostaining

Immunostaining with anti-Ki67 antibody (NeoMarkers, Fremont, CA) was performed to monitor cell proliferation. The number of Ki67-labeled cells was counted in six microscopic fields (10X) for each section.

Western blot. Liver protein (40 μg) was electrophoresed on SDS-polyacrylamide gels. Proteins from the gels were transferred to the polyvinylidene fluoride membranes. Anti-FGF21, CYCLIN D, CYCLIN E, CYP4A14, and β-actin (Santa Cruz, CA) antibodies were used for detection of proteins.

### Real-time quantitative polymerase chain reaction (qPCR)

Hepatic RNA isolated using TRIzol (Invitrogen, CA) was reverse transcribed to generate cDNA followed by amplification using the ABI Prism 7900HT sequence detection system (Applied Biosystems, CA). Hepatic mRNA levels were normalized based on hepatic *Gapdh* mRNA levels.

### Bile acid quantification

Methanol, water, and formic acid, all of LC/MS grade, were purchased from Fisher Scientific (Santa Clara, CA). CA, DCA, CDCA, UDCA, LCA, glycol and tauro derivatives were purchased from Steraloid Inc. (Newport, RI). Sample preparation was performed based on published methods [45]. The detection of hepatic BAs was carried out on a Prominence^TM^ UFLC system (Shimadzu, Kyoto) coupled to an API 4000 QTRAP^TM^ mass spectrometer (AB Sciex, CA) operated in the negative ionization mode. Chromatography was performed on a Kinetex C_18_ column (50 mm X 2.1 mm, 2.6 μm) maintained at 40°C preceded by a high pressure column prefilter. The mobile phase consisted of gradient of methanol delivered at a flow rate of 0.4 ml/min. MS parameters were described in our previous publication [46].

### Forced expression of FGF21 using recombinant adenovirus

Recombinant adenovirus expressing mouse FGF21 was purchased from Vector BioLabs (Vector Biolabs, PA). The adenoviral vector was transfected into a mammalian HEK293T cells to produce large-scale adenovirus [30]. Expression of FGF21 was achieved via tail vein injection [47]. Each mouse received 7.5 × 10^9^ particles/g body weight of Ad-Fgf21 or Ad-Control plasmid in 0.1 ml of saline before PH surgery [47].

### Chromatin immunoprecipitation (ChIP) PCR assays

ChIP assays were performed based on previous studies [48, 49]. Briefly, chromatin lysates were cleared before incubation with a ChIP-quality anti-PPARα antibody (Santa Cruz Biotechnology, Santa Cruz, CA). Antibodies to IgG (Santa Cruz Biotechnology, Santa Cruz, CA) and RNA Polymerase II (Millipore, Billerica, MA) were used as negative and positive controls, respectively. Samples were incubated with Dynase beads at 4°C overnight followed by de-crosslinking and purification. DNA fragments generated served as templates for Real-Time PCR using Power SYBR Green PCR Master Mix.

### Statistical Analysis

Data are given as mean ± SD. Statistical analysis was performed using Student's *t* test or one-way analysis of variance. Significance was defined by *p* < 0.05.
